# Age-Related Effects of COVID-19 Pandemic on Mechanical Reperfusion and 30-Day Mortality for STEMI: Results of the ISACS-STEMI COVID-19 Registry

**DOI:** 10.3390/jcm12062116

**Published:** 2023-03-08

**Authors:** Giuseppe De Luca, Magdy Algowhary, Berat Uguz, Dinaldo C. Oliveira, Vladimir Ganyukov, Oliver Busljetik, Miha Cercek, Lisette Okkels Jensen, Poay Huan Loh, Lucian Calmac, Gerard Roura i Ferrer, Alexandre Quadros, Marek Milewski, Fortunato Scotto D’Uccio, Clemens von Birgelen, Francesco Versaci, Jurrien Ten Berg, Gianni Casella, Aaron Wong Sung Lung, Petr Kala, José Luis Díez Gil, Xavier Carrillo, Maurits Dirksen, Victor Becerra Munoz, Michael Kang-yin Lee, Dafsah Arifa Juzar, Rodrigo de Moura Joaquim, Roberto Paladino, Davor Milicic, Periklis Davlouros, Nikola Bakraceski, Filippo Zilio, Luca Donazzan, Adriaan Kraaijeveld, Gennaro Galasso, Lux Arpad, Lucia Marinucci, Vincenzo Guiducci, Maurizio Menichelli, Alessandra Scoccia, Aylin Hatice Yamac, Kadir Ugur Mert, Xacobe Flores Rios, Tomas Kovarnik, Michal Kidawa, Josè Moreu, Vincent Flavien, Enrico Fabris, Iñigo Lozano Martínez-Luengas, Marco Boccalatte, Francisco Bosa Ojeda, Carlos Arellano-Serrano, Gianluca Caiazzo, Giuseppe Cirrincione, Hsien-Li Kao, Juan Sanchis Forés, Luigi Vignali, Helder Pereira, Stephane Manzo-Silberman, Santiago Ordoñez, Alev Arat Özkan, Bruno Scheller, Heidi Lehitola, Rui Teles, Christos Mantis, Ylitalo Antti, João António Brum Silveira, Cesar Rodrigo Zoni, Ivan Bessonov, Giuseppe Uccello, George Kochiadakis, Dimitrios Alexopulos, Carlos E. Uribe, John Kanakakis, Benjamin Faurie, Gabriele Gabrielli, Alejandro Gutierrez Barrios, Juan Pablo Bachini, Alex Rocha, Frankie C. C. Tam, Alfredo Rodriguez, Antonia Anna Lukito, Veauthyelau Saint-Joy, Gustavo Pessah, Andrea Tuccillo, Alfonso Ielasi, Giuliana Cortese, Guido Parodi, Mohammed Abed Burgadha, Elvin Kedhi, Pablo Lamelas, Harry Suryapranata, Matteo Nardin, Monica Verdoia

**Affiliations:** 1 Division of Cardiology, AOU Policlinico G Martino, 98124 Messina, Italy; 2 Department of Clinical and Experimental Medicine, University of Messina, 98122 Messina, Italy; 3 Division of Cardiology, Galeazzi-Sant’Ambrogio Hospital, 20157 Milan, Italy; 4 Division of Cardiology, Assiut University Heart Hospital, Assiut University, Asyut 71515, Egypt; 5 Division of Cardiology, Bursa City Hospital, 16110 Bursa, Türkiye; 6 Pronto de Socorro Cardiologico Prof. Luis Tavares, Centro PROCAPE, Federal University of Pernambuco, Recife 1235, Brazil; 7 Department of Heart and Vascular Surgery, State Research Institute for Complex Issues of Cardiovascular Diseases, 653002 Kemerovo, Russia; 8 University Clinic for Cardiology, Medical Faculty, Ss’ Cyril and Methodius University, 1000 Skopje, North Macedonia; 9 Department of Cardiology, Medical Center Ljubljana, 1000 Ljubljana, Slovenia; 10 Division of Cardiology, Odense Universitets Hospital, 5000 Odense, Denmark; 11 Department of Cardiology, National University Hospital, Singapore 119074, Singapore; 12 Clinic Emergency Hospital of Bucharest, 010001 Bucharest, Romania; 13 Interventional Cardiology Unit, Heart Disease Institute, Hospital Universitari de Bellvitge, 08016 Barcelona, Spain; 14 Instituto de Cardiologia do Rio Grande do Sul, Porto Alegre 90000-00, Brazil; 15 Division of Cardiology, Medical University of Silezia, 40-002 Katowice, Poland; 16 Division of Cardiology, Ospedale del Mare, 80147 Napoli, Italy; 17 Department of Cardiology, Medisch Spectrum Twente, Thoraxcentrum Twente, 7541 Enschede, The Netherlands; 18 Division of Cardiology, Ospedale Santa Maria Goretti Latina, 04100 Latina, Italy; 19 Division of Cardiology, St Antonius Hospital, 3434 Nieuwegein, The Netherlands; 20 Division of Cardiology, Ospedale Maggiore Bologna, 40100 Bologna, Italy; 21 Department of Cardiology, National Heart Center, Singapore 169609, Singapore; 22 University Hospital Brno, Medical Faculty of Masaryk University Brno, 60200 Bohunice, Czech Republic; 23 Cardiology, H. Universitario y Politécnico La Fe, 46001 Valencia, Spain; 24 Hospital Germans Triasi Pujol, 8918 Badalona, Spain; 25 Division of Cardiology, Northwest Clinics, 1811 Alkmaar, The Netherlands; 26 Hospital Clínico Universitario Virgen de la Victoria, 290000 Malaga, Spain; 27 Department of Cardiology, Queen Elizabeth Hospital, University of Hong Kong, Hong Kong; 28 Department of Cardiology and Vascular Medicine, University of Indonesia National Cardiovascular Center “Harapan Kita”, Jakarta 11402, Indonesia; 29 Instituto de Cardiologia de Santa Catarina Praia Comprida, São José 88100-000, Brazil; 30 Division of Cardiology, Clinica Villa dei Fiori, 80011 Acerra, Italy; 31 Department of Cardiology, University Hospital Centre, University of Zagreb, 10000 Zagreb, Croatia; 32 Invasive Cardiology and Congenital Heart Disease, Patras University Hospital, 26221 Patras, Greece; 33 Center for Cardiovascular Diseases, 6000 Ohrid, North Macedonia; 34 Division of Cardiology, Ospedale Santa Chiara di Trento, 38014 Trento, Italy; 35 Division of Cardiology, Ospedale ”S. Maurizio”, 39100 Bolzano, Italy; 36 Division of Cardiology, UMC Utrecht, 3584 Utrecht, The Netherlands; 37 Division of Cardiology, Ospedale San Giovanni di Dio e Ruggid’Aragona, 84070 Salerno, Italy; 38 Maastricht University Medical Center, 6229 HX Maastricht, The Netherlands; 39 Division of Cardiology, Azienda Ospedaliera “Ospedali Riuniti Marche Nord”, 61121 Pesaro, Italy; 40 Division of Cardiology, AUSL-IRCCS Reggio Emilia, 42121 Reggio Emilia, Italy; 41 Division of Cardiology, Ospedale “F. Spaziani“, 03100 Frosinone, Italy; 42 Division of Cardiology, Ospedale “Sant’Anna”, 44121 Ferrara, Italy; 43 Department of Cardiology, Hospital Bezmialem Vakıf University, 34093 Istanbul, Türkiye; 44 Division of Cardiology, Faculty of Medicine, Eskisehir Osmangazi University, 02640 Eskisehir, Türkiye; 45 Complexo Hospetaliero Universitario La Coruna, 15001 La Coruna, Spain; 46 University Hospital Prague, 12808 Prague, Czech Republic; 47 Central Hospital, Medical University of Lodz, 90-008 Lodz, Poland; 48 Division of Cardiology, Complejo Hospitalario de Toledo, 45001 Toledo, Spain; 49 Division of Cardiology, Center Hospitalier Universitaire de Lille, 59000 Lille, France; 50 Azienda Ospedaliero, Universitaria Ospedali Riuniti Trieste, 34142 Trieste, Italy; 51 Division of Cardiology, Hospital Cabueñes, 33201 Gijon, Spain; 52 Division of Cardiology, Ospedale Santa Maria delle Grazie, 80078 Pozzuoli, Italy; 53 Division of Cardiology, Hospital Universitario de Canarias, 38001 Santa Cruz de Tenerife, Spain; 54 Division of Cardiology, Hospital Puerta de Hierro Majadahonda, 28222 Madrid, Spain; 55 Division of Cardiology, Ospedale “G Moscati”, 81031 Aversa, Italy; 56 Division of Cardiology, Ospedale Civico Arnas, 90100 Palermo, Italy; 57 Cardiology Division, Department of Internal Medicine, National Taiwan University Hospital, 8865, Taipei 600, Taiwan; 58 Division of Cardiology, Hospital Clinico Universitario de Valencia, 46010 Valencia, Spain; 59 Interventional Cardiology Unit, Azienda Ospedaliera Sanitaria, 43121 Parma, Italy; 60 Cardiology Department, Hospital Garcia de Orta, Pragal, 2805-267 Almada, Portugal; 61 Division of Cardiology, CHU Lariboisière, AP-HP, Paris VII University, INSERM UMRS 942, 75010 Paris, France; 62 Instituto Cardiovascular de Buenos Aires, 6302, Buenos Aires C1428 CABA, Argentina; 63 Cardiology Institute, Instanbul University, 34000 Istanbul, Türkiye; 64 Division of Cardiology, Clinical and Experimental Interventional Cardiology, University of Saarland, 66421 Saarland, Germany; 65 Division of Cardiology, Oulu University Hospital, 90220 Oulu, Finland; 66 Division of Cardiology, Hospital de Santa Cruz, CHLO-Nova Medical School, 1000 Lisbon, Portugal; 67 Division of Cardiology, Kontantopoulion Hospital, 10431 Athens, Greece; 68 Division of Cardiology, Heart Centre Turku, 20521 Turku, Finland; 69 Division of Cardiology, Hospital de Santo António, 4099-001 Porto, Portugal; 70 Department of Teaching and Research, Instituto de Cardiología de Corrientes “Juana F. Cabral”, Corrientes W3400CDS, Argentina; 71 Tyumen Cardiology Research Center, 625026 Tyumen, Russia; 72 Division of Cardiology, Ospedale “A. Manzoni”, 23900 Lecco, Italy; 73 Iraklion University Hospital, 70001 Crete, Greece; 74 Division of Cardiology, Attikon University Hospital, 10431 Athens, Greece; 75 Division of Cardiology, Universidad UPB-CES, Medellin 050001, Colombia; 76 Division of Cardiology, Alexandra Hospital, 10431 Athens, Greece; 77 Division of Cardiology, Groupe Hospitalier Mutualiste de Grenoble, 38000 Grenoble, France; 78 Interventional Cardiolgy Unit, IRCCS INRCA, 60131 Ancona, Italy; 79 Division of Cardiology, Hospital Puerta del Mar, 11001 Cadiz, Spain; 80 Instituto de Cardiologia Integral, Montevideo 11700, Uruguay; 81 Department of Cardiology and Cardiovascular Interventions, Instituto Nacional de Cirugía Cardíaca, Montevideo 11700, Uruguay; 82 Department of Cardiology, Queen Mary Hospital, University of Hong Kong, Hong Kong; 83 Division of Cardiology, Otamendi Hospital, Buenos Aires 1001, Argentina; 84 Heart Center Siloam Lippo Village Hospital, Cardiovascular Department Pelita Harapan University, Tangerang 15810, Indonesia; 85 Center Hospitalierd’Antibes Juan Les Pins, 06600 Antibes, France; 86 Division of Cardiology, Hospital Cordoba, Cordoba 5000, Argentina; 87 Department of Statistical Sciences, University of Padova, 35121 Padova, Italy; 88 Cardiology, Azienda Ospedaliera Lavagna, 16033 Lavagna, Italy; 89 Division of Cardiology, Blida University Hospital, Blida 0900, Algeria; 90 Division of Cardiology, Hopital Erasmus, Universitè Libre de Bruxelles, 1050 Bruxelles, Belgium; 91 Division of Cardiology, Radboud University Medical Center, 6525 Nijmegen, The Netherlands; 92 Department of Internal Medicine, Ospedale Riuniti, 25121 Brescia, Italy; 93 Division of Cardiology, Ospedale degli Infermi, ASL, 13900 Biella, Italy

**Keywords:** ageing, ST-segment elevation myocardial infarction, COVID-19

## Abstract

Background: The constraints in the management of patients with ST-segment elevation myocardial infarction (STEMI) during the COVID-19 pandemic have been suggested to have severely impacted mortality levels. The aim of the current analysis is to evaluate the age-related effects of the COVID-19 pandemic on mechanical reperfusion and 30-day mortality for STEMI within the registry ISACS-STEMI COVID-19. Methods: This retrospective multicenter registry was performed in high-volume PPCI centers on four continents and included STEMI patients undergoing PPCI in March–June 2019 and 2020. Patients were divided according to age (< or ≥75 years). The main outcomes were the incidence and timing of PPCI, (ischemia time longer than 12 h and door-to-balloon longer than 30 min), and in-hospital or 30-day mortality. Results: We included 16,683 patients undergoing PPCI in 109 centers. In 2020, during the pandemic, there was a significant reduction in PPCI as compared to 2019 (IRR 0.843 (95%-CI: 0.825–0.861, *p* < 0.0001). We found a significant age-related reduction (7%, *p* = 0.015), with a larger effect on elderly than on younger patients. Furthermore, we observed significantly higher 30-day mortality during the pandemic period, especially among the elderly (13.6% vs. 17.9%, adjusted HR (95% CI) = 1.55 [1.24–1.93], *p* < 0.001) as compared to younger patients (4.8% vs. 5.7%; adjusted HR (95% CI) = 1.25 [1.05–1.49], *p* = 0.013), as a potential consequence of the significantly longer ischemia time observed during the pandemic. Conclusions: The COVID-19 pandemic had a significant impact on the treatment of patients with STEMI, with a 16% reduction in PPCI procedures, with a larger reduction and a longer delay to treatment among elderly patients, which may have contributed to increase in-hospital and 30-day mortality during the pandemic.

## 1. Background

Over 100 million cases of COVID-19, and more than 2 million deaths have been reported worldwide, leading to a severe commitment for the healthcare systems [1]. The conversion and occupation of many clinical units for COVID-19 patients led to the suspension of elective procedures and treatment of chronic conditions, whilst the maintenance of services for the management of urgent conditions, such as acute coronary syndromes, required to be preserved. Nevertheless, several previous reports showed a reduction in the number of treated acute coronary cases, accounted for by the fear of contagion preventing patients’ presentation at hospital [2,3,4,5,6,7,8,9,10]. An additional observation was the prolonged time from symptom onset to treatment [11,12,13], secondary to the oversaturation of the emergency departments, that contributed to explaining the higher mortality among STEMI patients observed in 2020.

Elderly patients, due to the higher prevalence of comorbidities, are those mostly fragile patients who could have been more largely affected by the pandemic, especially when presenting with ST-segment elevation myocardial infarction.

The International Study on Acute Coronary Syndromes–ST-elevation myocardial infarction (ISACS-STEMI) COVID-19 registry provided a snapshot of the treatment and outcomes of STEMI patients treated by primary angioplasty during the COVID-19 pandemic. The current analysis aimed to evaluate the age-related effects of the COVID-19 pandemic on mechanical reperfusion and 30-day mortality for STEMI within the registry.

## 2. Study Design and Population

This is a large-scale retrospective multicenter registry promoted by the Eastern Piedmont University, Novara, Italy. The initial planning was to include European primary PCI centers [9] but the study was subsequently extended to several other regions on different continents (Latin America, Southeast Asia and North Africa). Included centers were required to perform more than 120 primary PCI/year (with expected average > 10/month), with the STEMI caseload not expected to undergo a planned reorganization of the STEMI network. The initial inclusion period was of 2 months (from 1 March to 30 April) but was subsequently prolonged to 30 June 2020. The data were compared with those retrospectively collected during the same months of 2019 (from 1 March to 30 June).

Inclusion criteria: STEMI treated by primary angioplasty (including mechanical reperfusion for failed thrombolysis).

Data Collection: Anonymized data were collected through a dedicated CRF. Each center identified a local Principal Investigator. Demographic, clinical and procedural data, including total ischemia and door-to-balloon time, referral to primary PCI facility, COVID-19 positivity, PCI procedural data, and in-hospital mortality were recorded. Data were centralized and managed at Eastern Piedmont University.

Statistics. Data were analyzed using SPSS Statistics Software 23.0 (IBM SPSS Inc., Chicago, IL, USA) and R software (version 3.6.2, R Core Team, http://www.R-project.org, accessed on 24 June 2021) by an independent statistician (GC). Quantitative variables were described using median and interquartile range. Mean and confidence intervals were obtained assuming Poisson distributions for count data. Incidence rate ratio (IRR) was defined as the ratio between count data in 2020 and count data in 2019. Data were normalized for the different sizes of the national populations and for the possibly different time period of observation, and we considered the number of STEMI per million of residents in the corresponding population in a year (https://knoema.com/atlas/topics/Demographics/Age/Population-aged-75-years, accessed on 24 June 2021). Poisson regression models (with log link function) were applied to compare the incidence rates of primary PCI per million residents per year in 2020 with the same rate in 2019, correcting for possible impact of major risk factors [14]. Details are described in the Supplementary Materials (Section S1.1). Analyses were also conducted according to major European geographic areas (see Supplementary Materials) and subgroups of patients, according to age, gender, diabetes and hypertension.

A subsequent analysis was based on individual patient data, which were grouped according to the year of the intervention (2019 vs. 2020). Absolute frequencies and percentages were used for qualitative variables. ANOVA or Mann–Whitney and chi-square tests were used for continuous and categorical variables, respectively. Normal distribution of continuous variables was tested by the Kolmogorov–Smirnov test.

Multivariable logistic regression analyses were performed to identify the impact of the year of intervention on time delays and mortality after adjustment for baseline confounding factors between the two groups. All significant variables (set at a *p*-value < 0.1) were entered “in block” into the model. A *p* < 0.05 was considered statistically significant. The data coordinating center was established at the Eastern Piedmont University.

Sample size calculation. In view of the observational nature of this registry, no sample size calculations or statistical power analysis were performed.

## 3. Results

A total of 109 centers from four continents (Europe = 90; Latin America = 10; Southeast Asia = 7; North Africa = 2) participated (Table S1), leading to the inclusion of 16,674 STEMI patients, of whom 9044 patients were admitted in 2019 and 7630 patients in 2020. A total of 3178 patients were elderly (19.1% of the total population), with a similar proportion in both 2019 and 2020.

**Figure 1:**
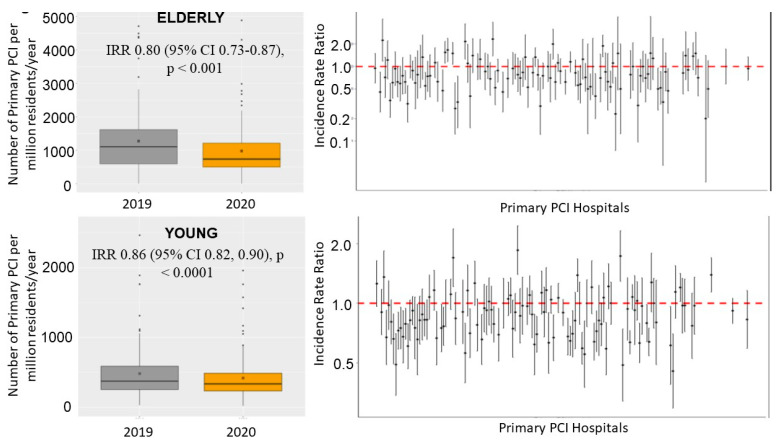
Box–and–whisker plot (on the left) showing the number of STEMI patients treated by mechanical reperfusion per million inhabitants/year in 2019 and 2020. The right graph shows the incidence rate ratio with 95% confidence interval across each center.

The number of STEMI patients treated percutaneously per million residents showed a consistent reduction, on average, from 559 (95% CI 514–607) in 2019 to 477 (95% CI 435–522) in 2020. (Figure 1 and Figures S1–S3). The incidence rate ratio (IRR) was 0.843 (95% CI 0.825–0.861, *p* < 0.0001), showing a significant reduction of 15.7% in the number of STEMI cases from 2019 to 2020.

We found a significant age-related reduction (7%, *p* = 0.015), with a larger effect in the elderly than in younger patients. Among elderly patients, the number of STEMI cases treated percutaneously per million residents had a consistent reduction, on average, from 1384 (95% CI 1312–1459) in 2019 to 1099 (95% CI 1035–1166) in 2020 (incidence rate ratio (IRR) 0.80 (95% CI 0.73–0.87), *p* < 0.001) (Figure 1 and Figure S1). A significant heterogeneity was observed across the centers (IRR had high variability between centers measured by std error = 0.35, ANOVA chi-square test for random vs. fixed effect Poisson model: *p* < 0.001) (Figure 1).

The number of STEMI cases treated percutaneously per million residents had a consistent reduction, on average, from 484 (95% CI 442–529) in 2019 to 420 (95% CI 381–462) in 2020 in younger patients, a less marked reduction (IRR was 0.856 (95% CI 0.82–0.90, *p* < 0.0001) as compared to elderly patients (Figure 1 and Figure S2). A significant heterogeneity was observed across centers (IRR had high variability between centers measured by a std error = 0.22, ANOVA chi-square test for random vs. fixed effect Poisson model: *p* < 0.001) (Figure 1).

The heterogeneity across centers was not related to the incidence of COVID-19 disease, nor to COVID-19-related mortality (Figures S3–S6). In fact, in both elderly and young patients, the reduction in STEMI procedures was not associated with the national number of COVID-19-positive patients, at either 30th of April (elderly: r = −0.075, *p* value = 0.438; young: r = 0.027, *p* value = 0.784) or 30th of June (elderly r = −0.028, *p* value = 0.773, Figure S3; young: r = 0.111, *p* value 0.25, Figure S4), nor with the national number of COVID-19-related deaths at 30th of April (elderly: r = −0.070, *p* value = 0.467; young: r = −0.002, *p* value = 0.98) or 30th of June (elderly: r = −0.120, *p* value = 0.221, Figure S5; young r = −0.017, *p* value = 0.863, Figure S6). Almost all participating continents had a reduction in STEMI cases (Figures S7–S10), that was significant only for European centers, whereas a larger reduction was observed in the young rather than elderly patients in North Africa.

Furthermore, we used Poisson regression to investigate the reduction in STEMI in subgroups of subjects in both elderly and young patients, by gender, hypertension, diabetes and smoking. We found a significant difference in this reduction between smokers (IRR = 0.85 (95% CI 0.80, 0.90), *p* < 0.0001) and non-smokers in young (IRR 0.78 (95% CI 0.73, 0.82) < 0.0001) (Figure S11) (*p* int = 0.024) but not in elderly patients (IRR 0.78 (95% CI 0.73, 0.82) < 0.0001) (Figure S12). No significant interaction was found for other variables (Figures S11–S14).

## 4. Baseline Demographic and Clinical Characteristics

Individual data analysis was restricted to 16,083 patients with complete demographic, clinical procedural and outcome data (complete cases: 96.4%), 8698 in 2019 and 7385 in 2020. Table 1 shows the baseline characteristics of elderly and young patients according to the year of intervention. No difference was observed in baseline characteristics.

**Figure 2:**
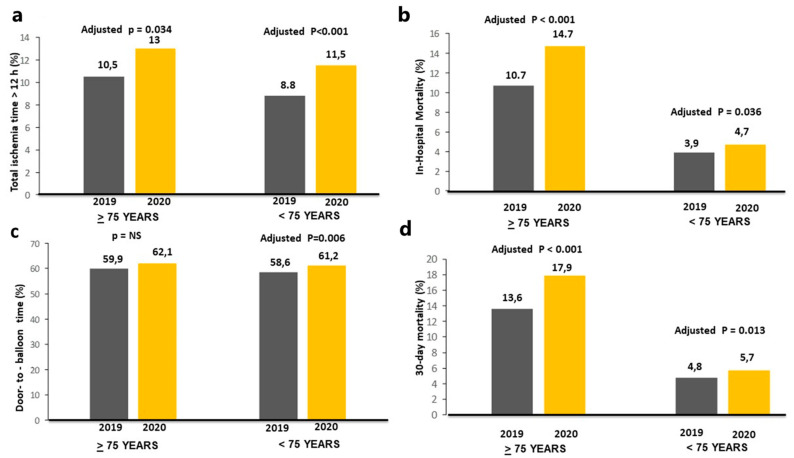
Bar graphs on the left side show the association between the year of intervention with time delays (ischemia time longer than 12 h, (**a**), upper-left graphs; door-to-balloon time longer than 30 min, (**c**), lower-left graphs). Bar graphs on the right side show the association between the year of intervention with in-hospital (**b**), upper-right graphs) and 30-day mortality (**d**), lower-right graphs).

As shown in Table 1, the COVID-19 pandemic was associated with a longer ischemia time, in both elderly and young patients, whereas a significantly longer door-to-balloon time was observed only in young patients (Figure 2).

The association between the COVID-19 pandemic and ischemia time longer than 12 h was confirmed, after correction for baseline clinical confounders in both the elderly (adjustment for geographic area, family history for CAD, radial access, door-to-balloon > 30 min and in-hospital RASI therapy; adjusted OR = 1.27 (1.02–1.59), *p* = 0.034), and young patients (adjustment for smoking, geographic area, previous PCI, door-to-balloon time > 30 min, DES, bivalirudin, mechanical support, in-hospital RASI therapy; adjusted OR = 1.35 (1.2–1.51, *p* < 0.001). No significant interaction was observed for major risk factors between young (gender, *p* = 0.19; diabetes, *p* = 0.25; hypertension, *p* = 0.89; smoking, *p* = 0.4), and elderly patients (gender, *p* = 0.36; diabetes, *p* = 0.12; hypertension, *p* = 0.57; smoking, *p* = 0.21).

The association between the COVID-19 pandemic and a door-to-balloon time longer than 30 min was confirmed after correction for baseline clinical confounders in young patients (adjustment for smoking, geographic area, previous PCI, ischemia time > 12 h, DES, bivalirudin, mechanical support, in-hospital RASI therapy; adjusted OR =1.11 (1.03–1.19), *p* = 0.006). No significant interaction was observed for major risk factors among young patients (gender, *p* = 0.46; diabetes, *p* = 0.32; hypertension, *p* = 0.12; smoking, *p* = 0.46).

No difference was observed in the rate of cardiogenic shock at presentation, infarct location, out-of-hospital cardiac arrest, or rescue procedures after failed thrombolysis.

The prevalence of SARS-CoV 2 positivity was low in both young and elderly patients (81 cases, 0.6% vs. 28 cases, 0.9%, *p* = 0.071).

## 5. Procedural Characteristics

Concerning procedural characteristics (Table 2), the use of DES and radial access were more frequent in 2020 (92.7% vs. 90.6%, *p* = 0.003) among young patients, whereas no differences were observed for other procedural variables.

## 6. In-Hospital and 30-Day Mortality

A significantly higher in-hospital mortality was observed in 2020 as compared to 2019 in both elderly (180 deaths, 10.7% vs. 200 deaths, 14.7%, OR (95% CI) = 1.43 (1.15–1.78), *p* < 0.001) and young patients (277 deaths, 3.9% vs. 281 deaths, 4.7%, OR (95% CI) = 1.19 (1.01–1.41), *p* = 0.043) (Figure 2).

The significantly poorer outcomes observed in STEMI patents treated in 2020 persisted after correction for all potential confounding factors in both elderly (adjustment for family history for CAD, geographic area, ischemia time, time, radial access, and in-hospital RASI) (adjusted OR (95% CI) = 1.64 (1.31–2.06), *p* < 0.001), and young patients (adjustment for smoking, geographic area, previous PCI, ischemia time, door-to-balloon time, DES, bivalirudin, mechanical support, in-hospital RASI therapy; adjusted OR (95% CI) = 1.22 (1.01–1.46), *p* = 0.036) (*p* interaction 0.12).

**Figure 3:**
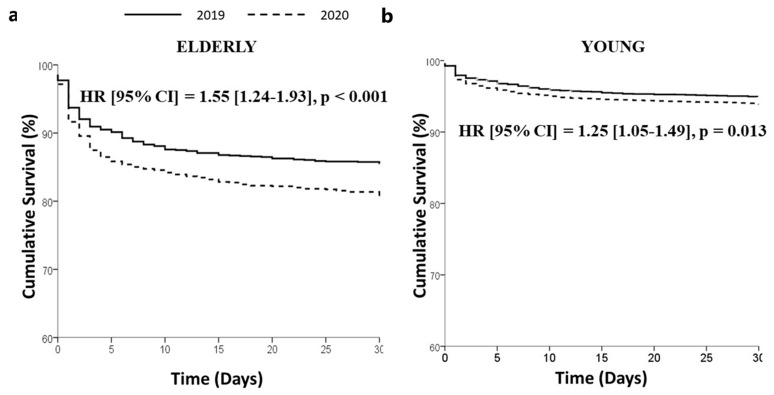
Kaplan–Meier survival curves of STEMI patients treated in 2019 and 2020 among elderly (**a**), left graph) and young patients (**b**), right graph).

Data on 30-day mortality were available in 14,303 (88.9%). Patients treated in 2020 had a significantly higher mortality in both elderly (201 deaths, 13.6% vs. 215 deaths 17.9%, adjusted HR (95% CI) = 1.55 (1.24–1.93), *p* < 0.001) and young patients (303 deaths, 4.8% vs. 308 death, 5.7%; adjusted HR (95% CI) = 1.25 (1.05–1.49), *p* = 0.013) (*p* interaction 0.24) (Figure 3).

SARS-CoV2 positivity was similarly associated with high mortality in both young (in hospital: 18.5% vs. 4.2%, OR (95% CI) = 5.2 (2.95–9.2), *p* < 0.001; 30-day: 26.5% vs. 5.1%, OR (95% CI) = 4.79 (2.92–7.85), *p* < 0.001) and elderly patients (in-hospital: 46.4% vs. 12.2%, OR (95% CI) = 6.3 (2.96–13.3), *p* < 0.001; 30-day: 58.3% vs. 15.2%, OR (95% CI) = 4.22 (2.47−7.18), *p* < 0.001).

## 7. Discussion

The ISACS-STEMI COVID-19 represents the largest registry worldwide, including more than 16,000 patients STEMI patients undergoing primary PCI during the COVID-19 pandemic, treated from March to June 2019 and 2020, and the first to provide data on 30-day mortality. This is the first report investigating the age-related impact of the COVID-19 pandemic on the management of STEMI. We found a significant reduction in the number of primary PCI procedures during the pandemic (in 2020) as compared to 2019, that was more marked in elderly patients. Although there was significant heterogeneity across the centers, it was not explained by the rate of either local or national deaths due to COVID-19. Furthermore, in-hospital and 30-day mortality were higher during the pandemic period, especially among elderly patients, likely reflecting the significantly longer ischemia time associated with impaired logistics and treatment during this challenging period.

Direct and indirect effects COVID-19 on cardiovascular disease and mortality have been identified [15].

Reports about the presence of inflammatory pathophysiological mechanisms, triggering plaque disruption and generating a pro-thrombotic milieu [16,17,18] supported an expected rise in the number of patients presenting with ACS during the pandemic.

Conversely, initial reports from small-sized registries showed a remarkable reduction in the number of acute coronary patients. These data were subsequently confirmed in a larger Chinese registry [8] and in European cohorts, including patients treated in March and April 2019–2020 [9,10].

Various factors are likely to have contributed to such a finding, with huge national and regional differences that could vary from −20 to −70% compared to pre-pandemic times [2,3,4,5,6,7,8,9,10]. It has been speculated that the need to shift healthcare resources for the treatment of COVID-19 patients, the isolation induced by the lock-down and the fear of contamination or burdening already overwhelmed clinical services could have prevented their presentation at hospital. Patients’ behavior may have contributed to increase morbidity and mortality, especially in STEMI patients in whom prolonged ischemia negatively impacted myocardial salvage, left-ventricular function, and both short and long-term survival [11,12,13]. Challenges in logistics for the ambulance system and emergency departments and the potential need to rule out potential COVID-19 positivity before admission may have contributed to the overall delay in treating patients with STEMI during the pandemic. Furthermore, effects associated with social distancing and isolation may also have played a role, including emotional stress, depression, and more sedentary lifestyle.

However, so far, no study has investigated the age-related impact of the COVID-19 pandemic on STEMI. In fact, elderly patients represent a fragile, high-risk population, with known atypical symptoms and more prolonged timing to diagnosis and treatment, higher thrombotic and bleeding risk and worse periprocedural outcome; factors that are expected to contribute to a higher susceptibility of this population to the deleterious direct and indirect effects of COVID-19 [19,20,21,22,23].

The data from the ISACS-STEMI COVID-19 registry, conducted in high-volume primary PCI centers on several continents (Europe, Latin America, Southeast Asia and North Africa provide relevant, reliable information for this controversial debate. Consistent with other small-sized registries and our previous report, we found a significant reduction in the number of STEMI patients undergoing mechanical reperfusion.

However, the reduction was significantly higher in elderly patients as compared to young patients. A major explanation for this finding is certainly the larger risk profile and presence of comorbidities among elderly patients. In fact, their frailty, lack of support from family members and the higher risk of mortality in the case of SARS-CoV2 infection, have certainly increased the fear of infection restraining them from contacting the emergency system even in the case of chest pain. Moreover, the initial misclassification of patients with dyspnea may have delayed access to the reperfusion therapies.

Notably, in step with previous reports, the reduction in STEMI patients undergoing mechanical revascularization was not consistent across all the centers. Additionally, it was not related to the local or national incidence of COVID-19 or rates of death due to COVID-19 in both groups of patients.

We cannot exclude local disparities among health care organizations and management of cardiovascular emergencies during the COVID-19 pandemic, which may have impacted on both the fear of contagion and the risk of out-of-hospital sudden death. Both factors may have contributed to the observed heterogeneity across centers.

We found that the COVID-19 pandemic was associated with a significantly longer ischemia time and rates of late presentation similarly occurring in young and elderly patients, whereas a higher rate of door-to-balloon time beyond 30 min was observed in both groups but statistically significant only in younger patients. This finding was presumably due to the larger sample size and statistical power of the young patients’ group. The longer door-to-balloon time may certainly be explained by organizational delays due to the specific COVID-19 protocols for screening patients and preparing equipment and personnel in the catheterization laboratory. Several additional factors may have played a role in the observed longer ischemia time during the COVID-19 pandemic, including both direct patients’ and emergency system-related delays, as previously described [24].

The longer delay to treatment contributes to the significantly overall higher mortality observed during this pandemic, as compared to 2019, that was confirmed after correction for major differences and, additionally, for COVID-19 positivity, in both young and elderly patients. We observed a more remarkable increase in mortality during the pandemic in the elderly, potentially explained by the larger thrombotic risk profile and fragility of elderly patients, but not ischemia time. However, we did not find a significant statistical interaction between the two groups in terms of mortality. Importantly, the COVID-19 positive population represented a very high-risk subgroup in both age groups, confirming recent reports by a smaller-sized study and our own group [9,25].

In light of the large vaccine campaign recently started worldwide and based on available data, it is extremely important that scientific societies and health authorities promote public campaigns in order to highlight the importance of the prompt recognition and response to the characteristic symptoms of acute myocardial infarction and the positive impact on the outcomes, especially among elderly patients.

## 8. Limitations

This study is limited by its retrospective design. It was conducted during a challenging pandemic emergency, and we expected to encounter missing data. Nevertheless, our main data analysis and conclusions are based on counts and, therefore, the overall cohort of patients was included. Furthermore, even in the analysis based on full individual patient data, this limitation and the potential risk of type II error was largely overcome by the high rate of complete cases (>95%) and the high statistical power due to the size of the study population. Finally, even though in the present registry of patients undergoing mechanical reperfusion, we did not find any difference in out-of-hospital cardiac arrest, we cannot exclude the possibility that the reduction in STEMI patients observed in 2020 may partly have resulted from higher rates of pre-hospital death due to longer delays to first medical contact, as was described during the COVID-19 pandemic [18,25]. Finally, primary PCI being the major reperfusion strategy worldwide, our registry was restricted to primary PCI centers. Therefore, we could not provide data on STEMI patients treated by thrombolysis.

## 9. Conclusions

The COVID-19 pandemic had a relevant impact on the treatment of patients with STEMI, with a significant reduction in primary PCI procedures, especially in elderly patients. We observed longer delays to treatment, which may have contributed to the increased in-hospital and 30-day mortality during this pandemic. Our data suggest that health authorities, supported by scientific societies, should take vigorous action to prevent patients from neglecting characteristic symptoms of an acute myocardial infarction, especially among elderly patients.

## Figures and Tables

**Table 1:** Baseline demographic and clinical characteristics.

	Elderly 2019 (*n* = 1682)	Elderly 2020 (*n* = 1365)	*p* Value	Young 2019 (*n* = 7016)	Young 2020 (*n* = 6020)	*p* Value
Age (median, IQR)	81 (77–85)	81 (77–85)	0.97	60 (52–66)	59 (52–66)	0.33 *
Male gender—*n* (%)	967 (57.5)	805 (59.0)	0.409	5604 (79.9)	4788 (79.5)	0.631
Medical History						
Hypertension—*n* (%)	1212 (72.1)	987 (72.3)	0.878	3533 (50.4)	3081 (51.2)	0.349
Diabetes mellitus—*n* (%)	490 (29.1)	390 (28.6)	0.734	1548 (22.1)	1384 (23.0)	0.207
Hypercholesterolemia—*n* (%)	721 (42.9)	611 (44.8)	0.294	2724 (38.8)	2297 (38.2)	0.434
Active smoker—*n* (%)	515 (30.6)	408 (29.9)	0.664	4314 (61.5)	3549 (59.0)	0.003
Family history of CAD—*n* (%)	200 (11.9)	128 (9.4)	0.026	1635 (23.3)	1335 (22.2)	0.126
Previous STEMI—*n* (%)	195 (11.6)	153 (11.2)	0.740	637 (9.1)	558 (9.3)	0.708
Previous PCI—*n* (%)	245 (14.6)	211 (15.5)	0.493	793 (11.3)	744 (12.4)	0.062
Previous CABG—*n* (%)	63 (3.7)	50 (3.7)	0.905	81 (1.2)	78 (1.3)	0.464
Geographic area			0.038			<0.001
Europe—*n* (%)	1476 (87.8)	1176 (86.2)		5507 (78.5)	4655 (77.3)	
Latin America—*n* (%)	89 (5.3)	106 (7.8)		541 (7.7)	614 (10.2)	
Southeast Asia—*n* (%)	92 (5.5)	67 (4.9)		614 (8.8)	520 (8.6)	
North Africa—*n* (%)	25 (1.5)	16 (1.2)		354 (5.0)	231 (3.8)	
Referral to Primary PCI Hospital						
Type			0.212			0.755
Ambulance (from community)—*n* (%)	848 (50.4)	720 (52.7)		3314 (47.2)	2856 (47.4)	
Direct access to hub—*n* (%)	439 (26.1)	319 (23.4)		2010 (28.6)	1745 (29.0)	
Transfer from spoke—*n* (%)	395 (23.5)	326 (23.9)		1692 (24.1)	1419 (23.6)	
Time delays						
Ischemia time, median (25–75th)	225 (140–375)	244 (150–430)	<0.0001	190 (120–345)	220 (130–402)	<0.0001 *
Total ischemia time			0.009			<0.001
<6 h—*n* (%)	1257 (74.7)	945 (69.2)		5365 (76.5)	4355 (72.3)	
6–12 h—*n* (%)	249 (14.8)	243 (17.8)		1035 (14.8)	972 (16.1)	
12–24 h—*n* (%)	113 (6.7)	110 (8.1)		424 (6.0)	441 (7.3)	
>24 h—*n* (%)	63 (3.7)	67 (4.9)		192 (2.7)	252 (4.2)	
Total ischemia time > 12 h—*n* (%)	176 (10.5)	177 (13.0)	0.032	616 (8.8)	693 (11.5)	<0.001 *
Door-to-balloon time, median (25–75th)	40 (25–70)	40 (26–74)	0.071	40 (25–62)	40 (25–70)	0.001 *
Door-to-balloon time			0.428			0.001
<30 min—*n* (%)	675 (40.1)	517 (37.9)		2904 (41.4)	2337 (38.8)	
30–60 min—*n* (%)	527 (31.3)	438 (32.1)		2318 (33.0)	1976 (32.8)	
>60 min—*n* (%)	480 (28.5)	410 (30.0)		1794 (25.6)	1707 (28.4)	
Door-to-balloon time > 30 min—*n* (%)	1007 (59.9)	848 (62.1)	0.205	4112 (58.6)	3683 (61.2)	0.003
Clinical Presentation						
Anterior STEMI—*n* (%)	803 (47.7)	654 (47.9)	0.925	3183 (45.4)	2806 (46.6)	0.155
Out-of-hospital cardiac arrest—*n* (%)	87 (5.2)	63 (4.6)	0.480	428 (6.1)	378 (6.3)	0.510
Cardiogenic shock—*n* (%)	170 (10.1)	148 (10.8)	0.509	455 (6.5)	395 (6.6)	0.550
Rescue PCI for failed thrombolysis—*n* (%)	82 (4.9)	57 (4.2)	0.358	523 (7.5)	437 (7.3)	0.670

* Mann–Whitney test; CAD = coronary artery disease; STEMI = ST-segment elevation myocardial infarction; PCI = percutaneous coronary intervention; CABG = coronary artery bypass graft.

**Table 2:** Angiographic and procedural characteristics.

	Elderly 2019 (*n* = 1682)	Elderly 2020 (*n* = 1365)	*p* Value	Young 2019 (*n* = 7016)	Young 2020 (*n* = 6020)	*p* Value
Radial Access (%)	1221 (72.6)	1031 (75.5)	0.066	5302 (75.6)	4714 (78.3)	<0.001
Culprit vessel			0.707			0.521
Left main—*n* (%)	38 (2.3)	24 (1.8)		103 (1.5)	87 (1.4)	
Left anterior descending artery—*n* (%)	805 (47.9)	629 (46.1)		3182 (45.4)	2742 (45.5)	
Circumflex—*n* (%)	206 (12.2)	183 (13.4)		1040 (14.8)	921 (15.3)	
Right coronary artery—*n* (%)	612 (36.4)	511 (37.4)		2648 (37.7)	2230 (37.0)	
Anterolateral branch—*n* (%)	4 (0.2)	2 (0.1)		21 (0.3)	14 (0.2)	
SVG—*n* (%)	17 (1.0)	16 (1.2)		20 (0.3)	26 (0.4)	
Proximal lesion location—*n* (%)						
In-stent thrombosis—*n* (%)	67 (4.0)	69 (5.1)	0.154	272 (3.9)	224 (3.7)	0.643
Multivesseldisease—*n* (%)	928 (55.2)	775 (56.8)	0.463	3308 (47.1)	2875 (47.8)	0.224
Preprocedural TIMI 0 flow—*n* (%)	1028 (61.1)	869 (63.7)	0.149	4738 (67.5)	4096 (68.0)	0.536
Thrombectomy—*n* (%)	242 (14.4)	180 (13.2)	0.34	1160 (16.5)	981 (16.3)	0.715
Stenting—*n* (%)	1491 (88.6)	1203 (88.1)	0.66	6507 (92.7)	5565 (92.4)	0.459
Drug-elutingstent—*n* (%)	1448 (86.1)	1176 (86.2)	0.958	6208 (88.5)	5422 (90.1)	0.004
Postprocedural TIMI 3 flow—*n* (%)	1500 (89.2)	1204 (88.2)	0.397	6530 (93.1)	5587 (92.8)	0.555
Gp IIb-IIIa inhibitors/cangrelor—*n* (%)	248 (14.7)	229 (16.8)	0.125	1505 (21.5)	1285 (21.3)	0.884
Bivalirudin—*n* (%)	4 (0.2)	3 (0.2)	0.918	30 (0.4)	15 (0.2)	0.083
Mechanical support—*n* (%)	57 (3.4)	54 (4.0)	0.406	189 (2.7)	197 (3.3)	0.052
*Additional PCI*			0.444			0.001
During the index procedure—*n* (%)	167 (9.9)	152 (11.1)		620 (8.8)	637 (10.6)	
Staged—*n* (%)	171 (10.2)	147 (10.8)		715 (10.2)	653 (10.8)	
DAPT therapy—*n* (%)	1647 (97.9)	1347 (98.7)	0.11	6946 (99.0)	5965 (99.1)	0.623
In-hospital RASI—*n* (%)	839 (49.9)	752 (55.1)	0.004	3787 (54.0)	3519 (58.5)	<0.001
In-hospital death—*n* (%)	180 (10.7)	200 (14.7)	0.001	277 (3.9)	281 (4.7)	0.043
Death—*n* (%)	201 (13.6)	215 (17.9)	0.002	303 (4.8)	308 (5.7)	0.03

TIMI = thrombolysis in myocardial infarction; DAPT = dual antiplatelet therapy.

## Data Availability

Data will be available upon request submitted to the steering committee for six months after publication.

## Supplementary Materials

The following supporting information can be downloaded at: https://www.mdpi.com/article/10.3390/jcm12062116/s1, Figure S1: This graph shows the results of Poisson regression analysis in the male population to study the relationship between the number of primary PCI per million of male residents/year in 2020 vs. the number in 2019.; Figure S2: This graph shows the results of Poisson regression analysis in the female population to study the relationship between the number of primary PCI per million of female residents/year in 2020 vs. the number in 2019.; Figure S3: This graph shows in the male population the absence of significant relationship between the Incidence Rate Ratio of each centre (on the log-scaled axis) and the number of national COVID-19 cases per million of male residents.; Figure S4: This graph shows in the female population the absence of significant relationship between the Incidence Rate Ratio of each centre (on the log-scaled axis) and the number of national COVID-19 cases per million of female residents.; Figure S5: This graph shows in the male population the absence of significant relationship between the Incidence Rate Ratio of each centre (on the log-scaled axis) and the number of national COVID-19 related deaths per million of male residents.; Figure S6. This graph shows in the female population the absence of significant relationship between the Incidence Rate Ratio of each centre (on the log-scaled axis) and the number of national COVID-19 related deaths per million of female residents.; Figure S7: Box-and-whisker plot showing the number of male STEMI patients treated by mechanical reperfusion per million of male residents/year in 2019 and 2020 across 4 continents.; Figure S8. Forest plots of the incidence rate ratio in the male population on the log-scaled axis with 95% confidence interval across each continent (1: Europe, 2: Latin America, 3: South East Asia, 4: North Africa).; Figure S9: Box-and-whisker plot showing the number of female STEMI patients treated by mechanical reperfusion per million of female residents/year in 2019 and 2020 across 4 continents.; Figure S10: Forest plots of the incidence rate ratio in the female population on the log-scaled axis with 95% confidence interval across each continent (1: Europe, 2: Latin America, 3: South East Asia, 4: North Africa).; Figure S11: Box-and-whisker plot showing the number of male STEMI patients treated by mechanical reperfusion per million of male residents/year in 2019 and 2020 (left graph) and the number of female STEMI patients treated by mechanical reperfusion per million of female residents/year in 2019 and 2020 (right graph) according to age (> or <75 years).; Figure S12: Box-and-whisker plot showing the number of male STEMI patients treated by mechanical reperfusion per million of male residents/year in 2019 and 2020 (left graph) and the number of female STEMI patients treated by mechanical reperfusion per million of female residents/year in 2019 and 2020 (right graph) according to hypertension.; Figure S13: Box-and-whisker plot showing the number of male STEMI patients treated by mechanical reperfusion per million of male residents/year in 2019 and 2020 (left graph) and the number of female STEMI patients treated by mechanical reperfusion per million of female residents/year in 2019 and 2020 (right graph) according to diabetes.; Figure S14: Box-and-whisker plot showing the number of male STEMI patients treated by mechanical reperfusion per million of male residents/year in 2019 and 2020 (left graph) and the number of female STEMI patients treated by mechanical reperfusion per million of female residents/year in 2019 and 2020 (right graph) according to smoking.; Figure S15: Forest plots of the incidence rate ratio in the male population on the log-scaled axis with 95% confidence interval according to major risk factors (Diabetes, Hypertension, Age and Smoking).; Figure S16: Forest plots of the incidence rate ratio in the female population on the log-scaled axis with 95% confidence interval according to major risk factors (Diabetes, Hypertension, Age and Smoking).; Table S1: Characteristics of participating centers.

## Abbreviations

PCI	Percutaneous coronary intervention
STEMI	ST-segment elevation myocardial infarction
DTB	Door-to-balloon time
IRR	Incidence rate ratio
ACS	Acute coronary syndrome
DES	Drug-eluting stent
RASI	Renin-angiotensin system inhibitors
